# There’s never been a better time to be a STEM educator

**DOI:** 10.1093/genetics/iyaf090

**Published:** 2025-05-30

**Authors:** Jason Williams

**Affiliations:** DNA Learning Center, Cold Spring Harbor Laboratory, One Bungtown Road, Cold Spring Harbor, NY 11724, USA

**Keywords:** STEM education, course-based research, genomics, nanopore sequencing

## Abstract

From its current vantage point, the future of US STEM education may appear bleak. Yet STEM education's strength and importance have never been greater, and evidence points to a bright future. This case can be made by drawing on the United State's identity as the world's most entrepreneurial nation. The optimistic outlook for STEM education is framed here through the lens of product-market fit—an economics concept describing how well-aligned products and market forces can generate self-sustaining demand. An analysis of these forces suggests that US STEM education has not only achieved this fit but surpassed it. The nation's strategic interests drive unmet demand for a well-prepared STEM workforce. Course-based research and inquiry-based teaching offer a superior educational model that can scale nationally. Life sciences, in particular, can combine broad student reach with low-cost DNA sequencing to create a multidisciplinary platform for education and research. As a grateful recipient of the Genetics Society of America's Elizabeth W. Jones Award, I reflect on how the Cold Spring Harbor Laboratory DNA Learning Center (DNALC) has operated at the intersection of these forces—developing infrastructure and approaches that are widely adopted and poised for expanded distribution. Meeting the nation's urgent need requires bold investment and broad engagement. By seizing this moment, we can make now the best time to be a STEM educator.

To persuade that STEM education deserves our nation's highest priority, I can’t offer any argument Vannevar Bush didn’t. Now 80 years old, *Science, The Endless Frontier* (Bush and Holt 2021) is essential reading in 2025. Each page of Bush's report to President Roosevelt is an early market analysis relevant to today's headlines and social media threads. Chapter 1 defends the “freedom of inquiry…under any plan for government support of science.” Chapter 2 argues university endowments are inadequate to the needs of funding research. Bush explains why the government—not private industry— must fund science, especially basic research. Quoting James Conant, Bush asserts: “the future of science in this country will be determined by our basic educational policy.”

Underinvesting in STEM education ignores clear demand for a scientifically literate workforce—a dangerous misalignment. Recent analysis finds that only 16% of US high school students are “STEM ready,” and just 3.2% ultimately enter the STEM workforce (Verma *et al*. 2022). This shortfall clashes with estimates that through 2032, STEM jobs will grow at least 7% faster per year than non-STEM jobs (National Science Board (US), 2024). Closing this gap will take fresh thinking and proven tools; both are within reach.

## Education supply chain failure: high quality but low delivery

The United States's failure to meet STEM education demand is not a flaw in the product, but in our ability to deliver it at scale. Over the past 20 years, STEM education has improved significantly as education research has shifted instruction away from lectures and memorization toward hands-on scientific practice. Mastery in sports comes from a playing field, not a textbook; effective STEM teaching borrows this principle to improve outcomes and expand access.

At the undergraduate level, course-based undergraduate research experiences (CUREs) are well-studied and high-impact. Similar inquiry-based approaches are also adaptable to secondary education. CUREs embed authentic research into the classroom—often over a semester or more—and are defined by 5 key features (Auchincloss *et al*. 2014): (i) engaging students in scientific practices such as developing hypotheses and models; (ii) enabling genuine discovery rather than repeating known outcomes; (iii) ensuring real-world relevance; (iv) fostering collaboration and communication; and (v) promoting iteration by helping students learn through overcoming challenges.

Students from all backgrounds who participate in CUREs show greater persistence in STEM, higher graduation rates, and a range of additional benefits (Dolan and Weaver 2021). Most importantly, CUREs solve a supply chain challenge: they scale authentic research to dozens—or even hundreds—of students per course, replacing 1-to-1 mentorship with a delivery model that meets current demand.

Barriers to implementing CUREs and inquiry-based teaching are well documented (DeChenne-Peters and Scheuermann 2022). Fixing the supply chain means enabling as many faculty as possible to overcome them. I’ve spent most of my career at the Cold Spring Harbor Laboratory DNALC, where I’ve had the privilege of helping build pedagogical infrastructure that lowers or removes barriers to STEM education.

Founded in 1988 by David Micklos, the DNALC develops tools and innovations that bring advanced, inquiry-driven science into classrooms across the United States and abroad. While 40,000 middle and high school students visit DNALC locations annually for field trips, its broader pedagogical infrastructure—including lab experiences, curricula, websites, software, educator networks, and partnerships—reaches over 500,000 learners each year.

DNALC faculty training equips educators to deliver research experiences. In 2011, we launched our DNA barcoding program, enabling students to use low-cost DNA extraction and sequencing protocols to identify plants, insects, and animals from their own backyards. The dnabarcoding101.org website provides protocols and curricular aids, highlights student programs (e.g. the *Urban Barcode Project* [Marizzi *et al*. 2018]), and showcases outputs such as published DNA sequences, specimen databases, and symposium posters. For analysis, the *DNA Subway* website offers cloud-based bioinformatics tools through a simple, classroom-friendly interface. The DNA barcoding CURE at James Madison University (Hyman *et al*. 2019)—a primarily undergraduate institution—reaches over 500 students annually, demonstrating DNALC resources can scale to support large introductory biology courses.

Optimizing educational infrastructure means fixing a key supply chain link: educator training. Our work has identified barriers to teaching advanced topics like bioinformatics (Williams *et al*. 2019) and advanced frameworks that help faculty adopt evidence-based practices, enrich curricula, and increase student participation (Handelsman *et al*. 2022).

As scientific discovery accelerates, the need to keep faculty up to date is urgent. Alongside a documented decline in opportunities for skill renewal (Micklos and Barone 2021), evidence shows that common professional development approaches are ineffective (Feldon *et al*. 2017). The “*Bicycle Principles*” (Williams *et al*. 2023)—a set of evidence-based recommendations for professionalizing short-format workshops—emerged from a proposal selected by the US National Science Foundation's 2026 Idea Machine Competition to identify national research priorities. Developed collaboratively by leading international training providers in the life sciences, the *Principles* reflect a consensus on evidence-based practice. High-quality short-format professional development can support STEM workforce members with decades of productivity ahead—many of whom are overlooked by reforms focused primarily on undergraduates.

A solid foundation is in place, but the success of infrastructure is tied to its invisibility, not its thrill. For real excitement, you need genomics.

## Genomics: STEM education's most exciting playground

The life sciences offer unmatched opportunities to engage students in authentic research, with broad interdisciplinary relevance and wide reach across student populations. CUREs now exist for every major area of biology, and for some time, we have called for national coordination to promote CUREs (Elgin *et al*. 2021) and provide clear pathways to workforce-relevant STEM learning.

Biology offers the widest on-ramp to STEM. At the high school level, it is the most widely taken STEM subject: 97% of students completed a biology course in both 2009 and 2019. Math follows—Geometry (92%) and Algebra (85%)—with chemistry trailing at 76% in 2019 (National Center for Education Statistics (US), 2022). In college, biology typically ranks first or second in STEM degree enrollment (National Center for Education Statistics (US), 2023).

Genomics offers a rich, multidisciplinary platform for research experiences. Most life sciences including molecular biology, genetics, ecology, physiology, and developmental biology can incorporate sequencing to explore core questions. CUREs involving the generation and analysis of DNA sequences are well-positioned for widespread adoption. With strong links to math, statistics, and artificial intelligence, genomics also opens broader pathways for students to develop computational skills.

Over the past 10 years, I’ve delivered more than a hundred workshops at nearly as many institutions across the United States and abroad—aspiring to be the “Johnny Appleseed” of genomics. Like the real John Chapman—who not only planted seeds but returned to nurture them—sharing genomics tools with faculty has meant both sparking initial interest and cultivating sustainable communities of practice.

DNA sequencing was once costly and time-consuming but advances in technology now allow high school students to perform the same experiments as experts (Reed *et al*. 2025). Handheld Oxford Nanopore MinION devices have disrupted the market by democratizing access to sequencing. A single MinION can produce up to 48 billion DNA bases—the equivalent of 15 human genomes—in 72 hours for under $2,000. With startup costs 30–50 times lower than other high-throughput platforms, MinIONs have brought advanced genomics within reach of most undergraduate and many high school classrooms. Thanks to its portability and increasing accuracy, Oxford Nanopore sequencing supports a wide range of applications—from de novo genome assembly to RNA sequencing and metagenomics (Zhang *et al*. 2024). Faculty can use this single, versatile platform to support diverse, student-led research.

It would be short-sighted not to imagine a future where every high school is a genome sequencing center. Students can now access the same technologies used in cutting-edge research—empowering them to pursue scientific questions, build workforce-ready skills, and deepen their engagement with science. Engaging even a fraction of the nation's 17 million high school students (National Center for Education Statistics (US), 2022) would dwarf the fewer than 100,000 life science graduate students enrolled each year (Fabina *et al*., 2023). Tapping into that scale of distributed innovation could position students as catalysts for biodiversity discovery, identifiers of emerging agricultural threats, or even patent holders of novel biochemical products.

To realize this vision, we’ve spent the past 3 years building the largest network of faculty bringing Oxford Nanopore sequencing into their classrooms, partnering directly with the company to expand access and create learning resources (Williams *et al*. 2025). One “Johnny Appleseed” anecdote perfectly captures the goal of scaling this innovation.

Last year, I was hosted at John F. Kennedy High School in Guam by Colette Beausoliel, a biology instructor and state Teacher of the Year, who gathered students from 5 of the island's high schools for an impromptu Nanopore sequencing workshop. In just 2 half-days, students—most of whom had never held a pipette—sequenced 16S rRNA from microbes collected from the bottoms of their shoes.

Six months later, at a high school research presentation at the National Institutes of Health, I was surprised to see one of those students again. With support from her mentors, she had acquired a Nanopore sequencer and launched an independent research project: sequencing the genome of an endemic fern.

Students can do this work. We simply need to give them the tools—and the infrastructure to scale it.

## Compounding returns, applied innovation

In entrepreneurship, success depends less on having the best idea and more on executing it better than anyone else. The same holds true in STEM education: we know what works—hands-on, research-based learning—but its impact depends on how effectively we deliver, support, and scale it.

The DNALC exemplifies this principle. Its outsized national and global impact began with a modest footprint: just a few teaching labs in its first 14 years were enough to connect thousands of local students and educators with the Nobel Prize-winning science of Cold Spring Harbor Laboratory. Today, the DNALC model has seeded a growing network—more than 20 science centers have been modeled after or directly launched by the Center. A newly developed licensing and partnership strategy now enables other institutions to replicate its proven delivery system.

One of the most exciting new chapters is unfolding in Puerto Rico, where a DNALC will open as part of the NSF's redevelopment of the former Arecibo Observatory. The Arecibo C3 STEM Center will combine DNALC programs with new hubs for human-centered computing and computational skills, multisensory teaching, education research, and advanced STEM engagement.

Arecibo C3's tagline, “Make STEM work for you,” reflects a delivery-focused mission: learn what works—through research that drives evidence-based practice; do what works—by scaling programs that build relevant skills; and share what works—by engaging entire communities in the economic and intellectual benefits of science.

For STEM education to truly succeed at scale, we must treat execution with the same rigor we apply to research.

## The enduring value of scientific truth

The unique opportunity of this moment calls for renewed investment in STEM education—not just in dollars, but in creativity and persistence. Just last week, I learned of the cancelation of NIH's Short-Term Program to Unlock Potential (STEP-UP)—the only NIH program offering formal research training and workforce development for high school students nationwide. STEP-UP is what brought me to Guam, and what brought the student mentioned earlier to NIH to share her work. That pipeline is now gone.

If product-market fit means having something the United States needs and a way to deliver it, STEM education has never been better positioned. We have the product: hands-on, evidence-based instruction. We have the market: students eager to tackle real problems with real tools. What remains to be seen is the foresight to provide investment and leadership. Policymakers must recognize this opportunity and act to fund, scale, and sustain it. Taxpayers must have the tools and opportunities to understand the value of the science they fund; researchers must treat professional science communication and education as essential—not optional.

Science educators carry 2 enduring commitments: to their students, and to the truth. Truth prevails over every attempt to hide, dilute, ignore, or destroy it. Students rely on us—not to tell them what to believe, but to equip them with the tools to reach conclusions worth believing.

Bets placed against the students or against the truth are guaranteed to fail. There has never been a better time to be a STEM educator.
